# Cognitive Behavioral Therapy Optimizing Post-Operative Outcomes Among Coronary Artery Bypass Graft Surgery Patients: A Systematic Review

**DOI:** 10.1192/bjo.2024.167

**Published:** 2024-08-01

**Authors:** Danya Ibrahim, Ibrahim H. Elkhidir, Zainab Mohammed, Da'ad Abdalla, Omer A. Mohammed

**Affiliations:** 1 Khartoum University, Faculty of Medicine, Khartoum, Sudan; 2 Internal medicine, Ribat University Hospital, Khartoum, Sudan

## Abstract

**Aims:**

This review aims to evaluate the lasting advantages of cognitive behavioral therapy (CBT) in alleviating anxiety and depression and improving overall health outcomes post-CABG.

**Methods:**

A comprehensive search across databases including Science Direct, PsycINFO, PubMed, Google Scholar, VHL, Cochrane, and Scopus was conducted up to October 2023. The study adhered to the Preferred Reporting Items for Systematic Reviews and Meta-Analyses (PRISMA) statement and Cochrane guidelines. Inclusion criteria involved randomized controlled trials reporting on CBT or CBT-based interventions tailored for CABG patients and control groups had usual care, with anxiety and depression symptoms, as well as quality of life, as primary outcomes. Outcome variations were analyzed through standard deviation, while efficacy was measured via confidence intervals. Evaluation of the intervention process included examining feasibility, adherence, acceptability, inclusion rates, safety, and tolerability.

**Results:**

Three randomized controlled trials including a pilot study in America and Denmark, encompassing a total of 286 patients, were reported in this review. The participants' pooled mean age was 63.19 years (intervention) and 63.9 years (control), the male-to-female ratio was approximately 2:1 [males n = 174 (intervention n = 101; control = 73) while females n = 91 (intervention n = 60; control n = 31)], and cardiac as well as non-cardiac comorbidities including psychiatric diagnosis have been reported. The array of therapies ranged from education on anxiety and depression management skills to a combination of CBT and supportive stress management (SSM), and psychoeducational interventions paired with physical therapy. Results indicated that both CBT and SSM led to improvements in anxiety and depression symptoms, accompanied by reduced hospital stays, decreased hopelessness, lower scores in dysmorphic mood and irritability, lowered perceived cognitive impairment and stress, and increased satisfaction with therapy. The cognitive behavioral therapy demonstrated commendable feasibility, acceptability, safety, and efficacy, with some concerns raised about potential issues of low adherence.

**Conclusion:**

This systematic review emphasizes the positive impact of CBT on depression, anxiety, and quality of life in individuals post-CABG surgery. Future studies should adopt standardized CBT protocols, comprehensively evaluating CBT's influence on overall patient prognosis, considering cardiovascular outcomes across diverse ethnic groups, exploring cost-effectiveness, and specific patient cohorts that could benefit the most from CBT interventions.

